# Neurobiologically realistic neural network enables cross-scale modeling of neural dynamics

**DOI:** 10.1038/s41598-024-54593-w

**Published:** 2024-03-01

**Authors:** Yin-Jui Chang, Yuan-I Chen, Hsin-Chih Yeh, Samantha R. Santacruz

**Affiliations:** 1 Biomedical Engineering, The University of Texas at Austin, Austin, TX USA; 2 Texas Materials Institute, The University of Texas at Austin, Austin, TX USA; 3 Institute for Neuroscience, The University of Texas at Austin, Austin, TX USA; 4 Electrical and Computer Engineering, The University of Texas at Austin, Austin, TX USA

**Keywords:** Dynamical systems, Network models

## Abstract

Fundamental principles underlying computation in multi-scale brain networks illustrate how multiple brain areas and their coordinated activity give rise to complex cognitive functions. Whereas brain activity has been studied at the micro- to meso-scale to reveal the connections between the dynamical patterns and the behaviors, investigations of neural population dynamics are mainly limited to single-scale analysis. Our goal is to develop a cross-scale dynamical model for the collective activity of neuronal populations. Here we introduce a bio-inspired deep learning approach, termed NeuroBondGraph Network (NBGNet), to capture cross-scale dynamics that can infer and map the neural data from multiple scales. Our model not only exhibits more than an 11-fold improvement in reconstruction accuracy, but also predicts synchronous neural activity and preserves correlated low-dimensional latent dynamics. We also show that the NBGNet robustly predicts held-out data across a long time scale (2 weeks) without retraining. We further validate the effective connectivity defined from our model by demonstrating that neural connectivity during motor behaviour agrees with the established neuroanatomical hierarchy of motor control in the literature. The NBGNet approach opens the door to revealing a comprehensive understanding of brain computation, where network mechanisms of multi-scale activity are critical.

## Introduction

Billions of individual neurons coordinate activity at multiple scales, either directly or indirectly, to drive behaviour such as motor preparation^1,2^, motor adaptation^3^, motor timing^4,5^, decision-making^6^, and working memory^7,8^. However, current techniques for capturing neural population dynamics are mainly limited by the single-scale analysis, typically with the simplified assumptions of linear^9^ or log-linear^10^ dynamics. While recurrent neural networks (RNNs) have been introduced to infer nonlinear latent dynamics that encode rich information giving rise to motor behavior^11^, we lack a broadly accepted approach to explore cross-level activity for a deeper understanding of system-level nonlinear neural mechanisms^12,13^. Since the brain exhibits computational structure across a variety of scales, from single neurons (micro-scale) to functional areas (meso-scale) and cortical networks (macro-scale), a tool that can uncover multi-scale dynamics is critically important for illuminating the mechanistic understanding of brain activity^14^.

Until recently, only a limited number of studies focused on cross- or multi-scale interactions in brain networks. For example, source localization (e.g., sphere head model^15^) aims to identify the brain areas or individual neurons generating the recorded electrical potentials such as electroencephalography^16^. However, the requirements of high-density recordings, unrealistic assumptions, and uncertainty on conductivity value^17^ limit the fidelity of experimental data. In addition, cross-level coupling (CLC)^18^ has shown evidence of cross-scale interactions between single neurons and oscillatory network activity. In contrast, no information about how the activity communicates across levels is provided. Recent work developed a generalized linear model-based method to reveal the directed interactions across spatiotemporal scales of brain activity^19^. Nevertheless, brain dynamics are characterized by nonlinear coupling among neuronal populations^20,21^. Linear model-based approach may fail to capture the associated nonlinearity in the multi-scale brain networks.

**Figure 1:**
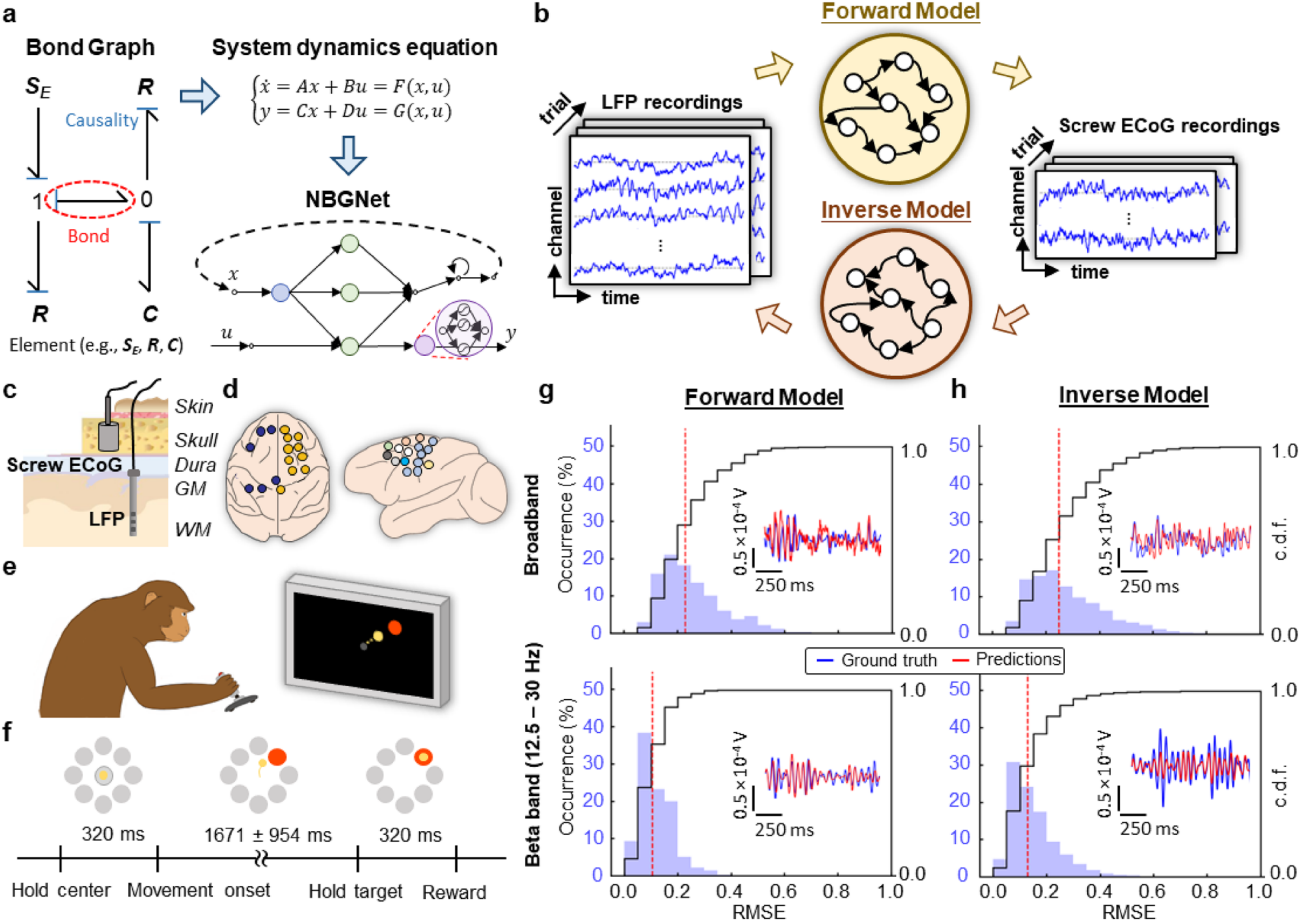
The NBGNet is a neurobiologically realistic recurrent neural network that utilizes nonlinear dynamics to model the translation between multi-scale brain activities. (**a**,**b**) Schematic overview of the NBGNet architecture for forward and inverse modeling between LFP and screw ECoG. Details are provided in the main text. (**c**) Spatial relationships between LFP and screw ECoG. (**d**) Screw ECoG signals were recorded across both hemispheres and LFP data was acquired from one hemisphere. Only 16 LFP channels were shown as a schematic. (**e**) Monkey performed a center-out reaching task using a joystick. (**f**) Schematic of protocol for the experiments. (**g**,**h**) Histogram and cumulative distribution function (c.d.f.) of RMSE in broadband and beta band (12.5–30 Hz) for forward model (**g**) and inverse model (**h**; red dashed line: median). The insets show the representative examples with the RMSE close to the red dashed line.

Successful modeling of multi-scale brain dynamics requires two challenges to be overcome: (1) a correct characterization of multi-scale interaction, and (2) a robust approach to approximate the nonlinearity embedded in the brain. The former can be addressed using a well-known modeling approach, termed the Bond Graph (BG), in the engineering field. The BG is a graphical approach widely used to model multi-domain dynamical systems (e.g., electrical, fluid, mechanical, magnetic, thermal, and hydraulic) via energy exchange^22^. BG allows a compact and explicit representation of the complex system and provides analogous applicability to different domains using the common constitutive relations: the element acting with the energy, the bond representing the energy transfer, and the causality depicting the government of the transfer (Fig. 1a). Such a graphical approach provides an easy way to connect and integrate the multi-domain system. For example, the BG can be utilized to model the system where the direct current (DC) motor converts the electrical energy into the mechanical energy to rotate a rotary plate. With the analogy between multi-domain and multi-scale modeling, we extend the BG approach to model multi-scale dynamical systems in brain networks, yielding a neurobiological-inspired state-space model with a priori knowledge of signal translations between multi-scale signals. The second challenge can be addressed with deep neural networks. Ultimately, combining both the BG and the deep neural networks, we leverage the NeuroBondGraph Network (NBGNet)^23^, a deep learning framework consisting of recurrent neural networks (RNNs) and multi-layer perceptrons (MLPs), to capture the temporal evolution and the nonlinearity of the system dynamics. Unlike source localization, incorporating neurobiological knowledge (specifically tissue electrical impedance) eliminates bias due to unrealistic assumptions (e.g., homogeneous tissue conductivity and ignorance of tissue capacitance). Compared to CLC, the NBGNet models the causal contributions which describe how individual and populations of neurons communicate in a cross-scale network. While purely data-driven methods, such as generalized linear models or black-box RNNs, may achieve similar performance, the NBGNet approach provides rigorous interpretability to evaluate both within- and cross-scale causal interactions.

The NBGNet model is universal in that it can be used for any combination of neural activity at different scales (or even the same scale) with the appropriate modification to the BG structure and its derived dynamic equations. To demonstrate the power of our approach, we employ two specific types of simultaneously recorded real neural data in this work. Namely, we use local field potentials (LFPs; total 157 channels in the spatial scale of 10^–4^ to 10^–5^ m) and signals recorded from intracranial “screw-type” macroelectrodes implanted in the skull (screw electrocorticography or screw ECoG^24,25^; total 16 channels in the spatial scale of 10^–2^ to 10^–4^ m) acquired from a rhesus macaque performing a simple motor task (Fig. 1b–f). Screw ECoG, rather than electroencephalography, is chosen due to its improved signal-to-noise ratio and stability. The structure of the NBGNet for these two particular data types is easily extended to other field potential signals, as well as spiking data with minor modifications.

We demonstrate that the NBGNet provides superior reconstruction accuracy with up to 11.1-fold decrease in root mean square error and 1.8- and 1.4-fold stronger similarity in time- and phase-domain compared to alternative methods. We show the NBGNet-derived causal interactions align well with the neuroanatomical hierarchy of motor control^26^, demonstrating the interpretability of the model structure. We further validate the capability of the NBGNet to capture and reconstruct single-trial low-dimensional neural dynamics. Behavioral variables can also be detected by NBGNet-predicted activity as accurately as using empirical measurements. Finally, we examine the stability of the NBGNet and reveal that the learned dynamical system maintains predictive power over more than 2 weeks without model retraining.

## Results

### Validation of NBGNet predictions using a center-out joystick task

We evaluate the prediction accuracy of the presented NBGNet by calculating the RMSE with the acquired broadband signals over 150 individual reach trials (Fig. 1g,h). Since the beta frequency band (12.5–30 Hz) is strongly implicated in motor behaviors^27,28^, we also examine the performance specifically within the beta band activity (Fig. 1g). Gated recurrent unit-based RNN (GRU-RNN) is utilized as baseline for quantitative comparison. For the forward solution, the NBGNet yielded 17% and 42% higher accuracy than GRU-RNN and sphere head model in trial-wise (RMSE = 0.12 ± 0.06 for NBGNet, 0.14 ± 0.06 for GRU-RNN, and 0.17 ± 0.07 for sphere head model; mean ± s.d. in the unit of 10^–4^ V), 7% and 53% in session-averaged comparison (RMSE = 0.15 for NBGNet, 0.16 for GRU-RNN, and 0.23 for sphere head model), respectively.

We also assess the capability of reconstructing LFP using inverse-NBGNet and screw ECoG recordings (Fig. 1h). Similarly, inverse-NBGNet outperforms GRU-RNN and sphere head model in both trial-wise (RMSE = 0.15 ± 0.10 for NBGNet, 0.17 ± 0.09 for GRU-RNN, and 1.68 ± 8.03 for sphere head model) and session-averaged comparisons (RMSE = 0.19 for NBGNet, 0.20 for GRU-RNN, and 2.42 for sphere head model). Interestingly, similarly small reconstruction error reveals that the inverse-NBGNet is able to transform the lower-dimensional screw ECoG into the higher-dimensional LFP.

### NBGNet outputs correlate with ground truth signals

**Figure 2:**
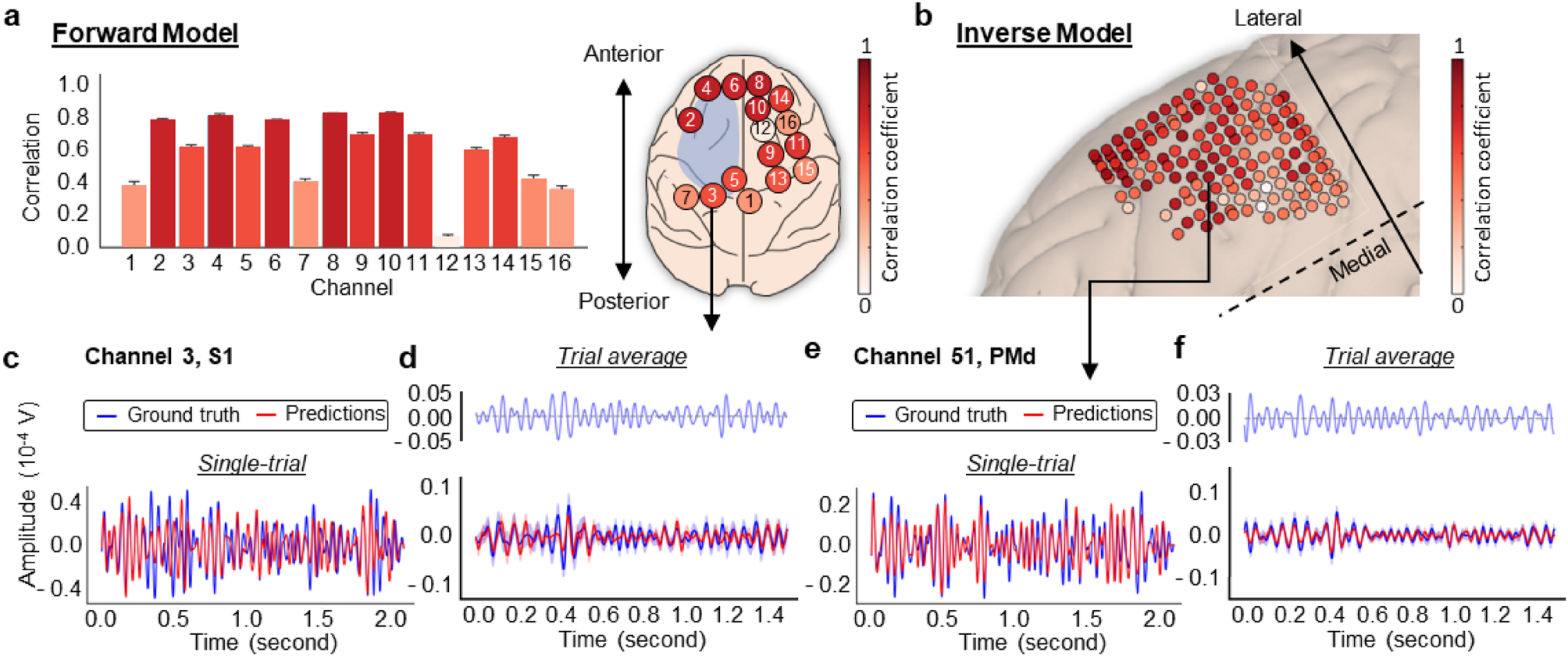
Cross-correlation analysis indicated the similarity between NBGNet inference and ground-truth recordings. (**a**) Average correlation coefficient across all the trials (error bars, s.d.; n = 150). Screw ECoG electrodes layout labeled with the channel number. Blue shaded area represents the coverage of LFP channels. (**b**) Same as (**a**) for the inverse model. A screw ECoG channels (3: S1) was selected for single trial-based comparison (**c**): ground truth (blue trace) versus model prediction (red trace) in the 3rd trial, and grand average-based comparison (**d**): ground truth (blue trace; mean ± s.e.m.) versus network output (red trace; mean ± s.e.m.) and the corresponding error trace (top). (**e**,**f**) Same as (**c**,**d**) for representative comparison for the inverse model (51: PMd).

Similarity of oscillation dynamics is an alternative approach to evaluate the integrity of predicted signals using cross-correlations computed on a single-trial single-channel basis. From the representative session (Fig. 2a,b), NBGNet-predicted signals from most of the channels are moderately to strongly correlated with the ground-truth signals (average correlation greater than 0.4^29,30^). A strong correlation (correlation coefficient > 0.6) is found on 63% of channels. Interestingly, due to movement-induced activation, channels in anterior brain regions exhibit greater correlation than those in posterior brain regions. The predicted screw ECoG matches well with the raw screw ECoG in both trial-wise (Fig. 2c) and session-averaged comparison (Fig. 2d). We note that the performance is relatively poor during 0.3–0.4 s when the subject is searching for the correct direction of cursor’s movement. However, the performance is better in the remainder of the time interval of interest when the direction of movement aligns with the target direction.

We also examine the correlation between the inverse-NBGNet-inferred and the ground-truth LFPs. Channel 29 (white matter) provides the highest correlation as 0.90 ± 0.07 (mean ± s.d.; Fig. 2e,f); whereas channel 82 (M1) exhibits no correlation (0.00 ± 0.30) due to an unexpectedly larger amplitude. However, the predicted signals on most of the other channels are moderately correlated with ground-truth activity. Strong correlations are found on 54% of channels. As the more lateral brain recording sites are also further away from the surface (compared with more medial regions) where screw ECoG was recorded, the channels in these regions show smaller correlations (Fig. 2b). In summary, the correlation analysis confirms the NBGNet’s ability to capture the beta-frequency dynamic features.

### Phase agreement in beta band during movement

**Figure 3:**
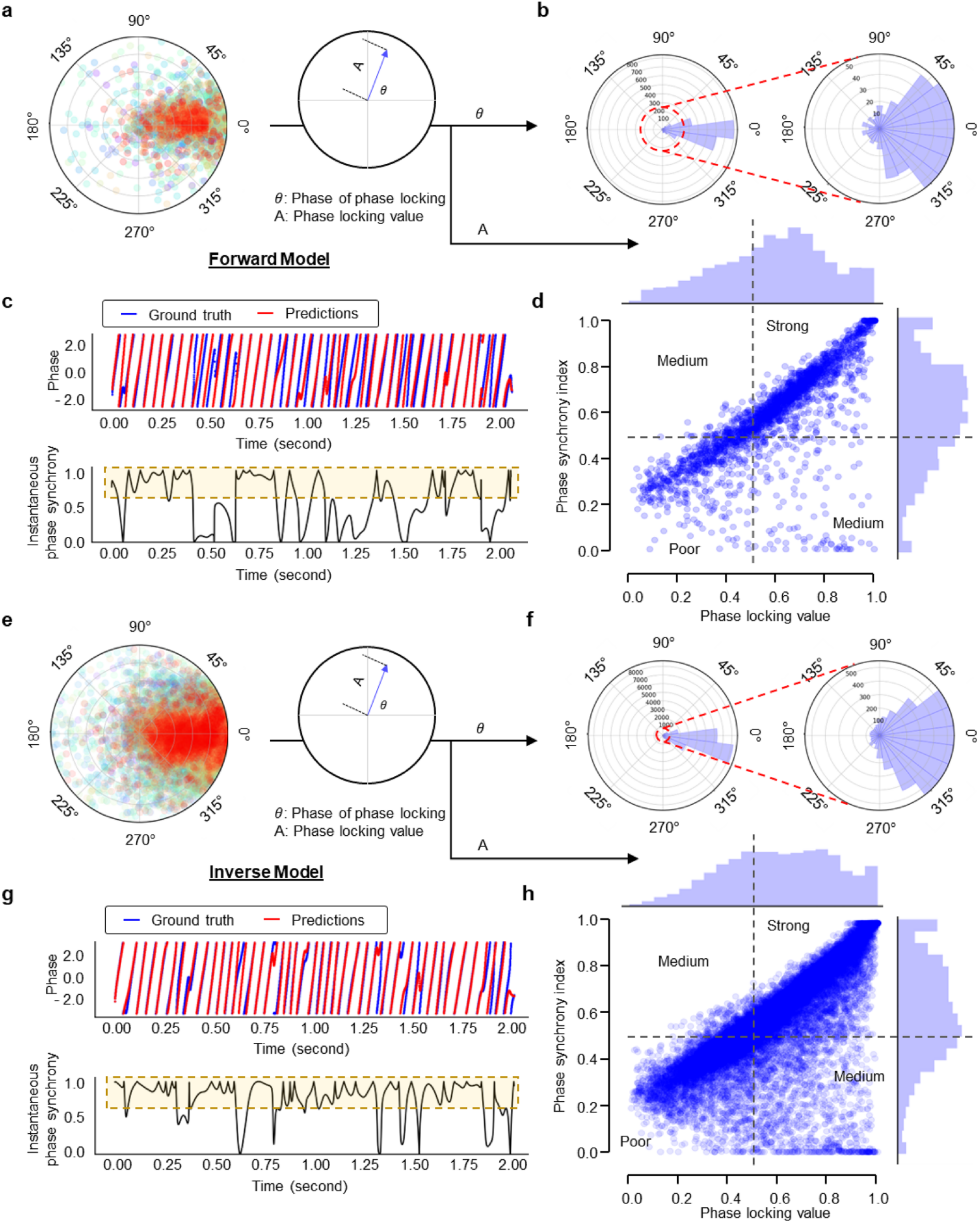
Strong phase synchrony between NBGNet estimations and the experimental recordings. (**a**) Polar plots of the mean phase difference averaging across time in each trial and each channel for the forward model (n = 2400). (**b**) Angular and corresponding zoom-in histogram of the phase of phase-locking derived from (**a**). (**c**) A screw ECoG channel (3: S1) was selected to demonstrate that NBGNet made predictions in sync with the ground truth in the 4th trial. The instantaneous phase of the ground truth (blue trace) and the model inference (red trace) at each timepoint (upper) were employed to obtain the instantaneous phase synchrony (lower; black trace) across the time. Yellow area showed a strong synchronization utilized to compute the phase synchrony index (PSI). (**d**) A scatter plot of phase analysis on each channel and each trial, respectively (n = 2400), revealing the expected and hidden relations between PSI and phase-locking value (PLV). Histograms of both PLV and PSI are represented on the *x* and *y* axes, respectively. 0.5 was set as thresholds for both PSI and PLV (black dashed line) to identify strong, medium, and poor synchrony regions. (**e**) Same as (**a**) for the inverse model (n = 23,550). (**f**) Same as (**b**) for the inverse model, where the histogram was derived from (**e**,**g**) Same as (**c**), where the chosen LFP channel for demonstration was the same as Fig. 2g,h. (**h**) Same as (**d**) for the inverse model (n = 23,550).

As phase-domain coherence is an important tool to determine the functional connectivity in brain networks, we examine whether the predicted and the recorded signals were phase-synchronized. Phase-locking value (PLV) has been widely used to measure the inter-trial variability of phase difference, where 1 represents no change in phase difference and 0 reflects the opposite case^31,32^. To assess the intra-trial variability, we adapt PLV by averaging the phase difference across the time rather than the trials (Fig. 3a,e). We also evaluate the phase of phase-locking to compare the average phase difference. Furthermore, to quantitively assess the phase similarity, instantaneous phase synchrony is applied to obtain the phase synchrony index (PSI) at each time point. If the phase difference seldom exceeds 45°, PSI is close to 1; it is close to zero otherwise. Forward-NBGNet-predicted signals are in sync with the ground truths (73% of average phase difference < 22.5°; average PLV = 0.59; average PSI = 0.59; Fig. 3b). Notably, the phase-synchronized predictions are generated in channel 3 (PSI = 0.51; Fig. 3c). We further assess the phase locking and phase synchrony simultaneously for each channel and each trial in the form of a scatter plot (Fig. 3d). Segmentation of the scatter plot enables us to study the further details. A larger fraction (74%) of predictions exhibit moderate or strong phase synchronization. We next evaluate the inverse-NBGNet’s inference of the synchronous LFPs. Similarly, the predictions are in sync with the ground-truth LFPs (75% of average phase difference < 22.5°; average PLV = 0.60; average PSI = 0.60; Fig. 3f). Notably, highly synchronized predictions at a representative channel are also observed (PSI = 0.83 for channel 51; Fig. 3g). Furthermore, approximately half of the predictions have strong synchronization (Fig. 3h). Our phase analysis comprehensively validates that the model predictions are phase-synchronized with the ground truth.

### NBGNet reveals cross-scale causal interactions among brain regions

**Figure 4:**
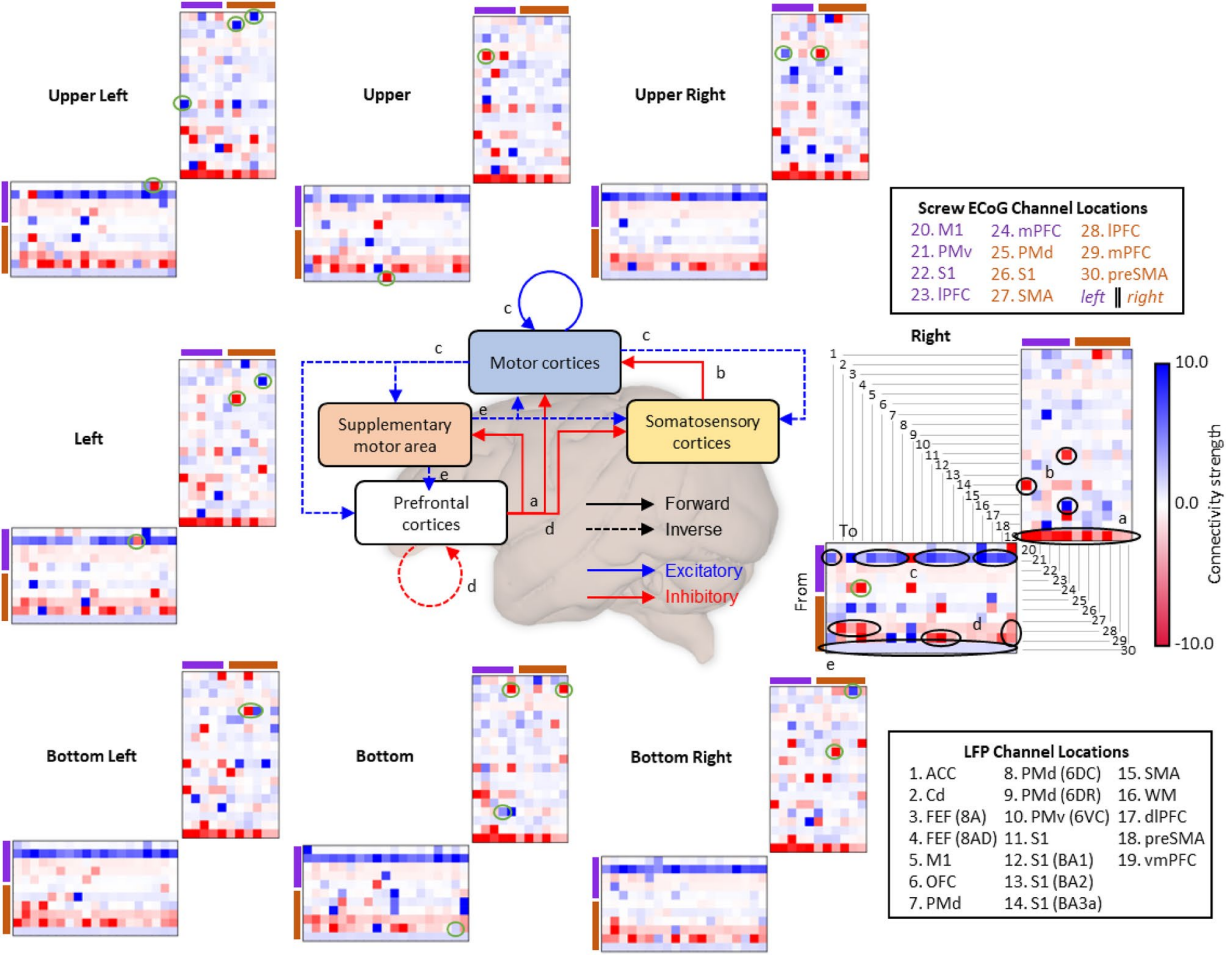
Bi-directional effectivity connectivity extracted from NBGNets exhibited unique and shared patterns in the center-out joystick task. The bi-directional effective connectivity for each target direction was obtained from the NBGNet’s parameters and was averaged over the trials reaching the same target. Each subfigure corresponds to a target position. The vertical axes represent the channels where the connection originates; the horizontal axes represent the channels where the connection contributes to. The shared patterns were indicated with black circles; the unique patterns were indicated with the green circles. The circuitry diagram (middle) depicts the hierarchical interactions between brain regions from the shared patterns of effective connectivity.

The complex coordination of brain functions, such as vision, motor preparation, and attention requires the control of causal interactions between areas^33^. Effective connectivity, which represents the influence that a neural system exerts over another^34^, is thus a powerful measure to evaluate the brain computations. In the NBGNet, we are able to derive the cross-scale effective connectivity that depicts how the latent states of sources change those of targets. Positive and negative connection strengths correspond to excitatory and inhibitory effects, respectively. The cross-scale effective connectivity exhibits patterns of visual feedback (unique to target position) and voluntary movement (shared across target position; Fig. 4) for different movement directions in the center-out joystick task. During rightward movement, a unique inverse connectivity from lateral prefrontal cortex to frontal eye field is observed, exhibiting a strong preference for contralateral visual space^35^. Furthermore, the identification of multiple shared causal interactions (e.g., prefrontal cortex-supplementary motor area, prefrontal cortex-motor cortex, and somatosensory cortex-motor cortex) over all the target directions aligns well with the abstraction of the hierarchical anatomy of the mammalian nervous system^26,36^. Thus, NBGNet-derived effective connectivity holds great potential to illuminate the cross-scale computations underlying brain functions.

### NBGNet captures latent dynamics

**Figure 5:**
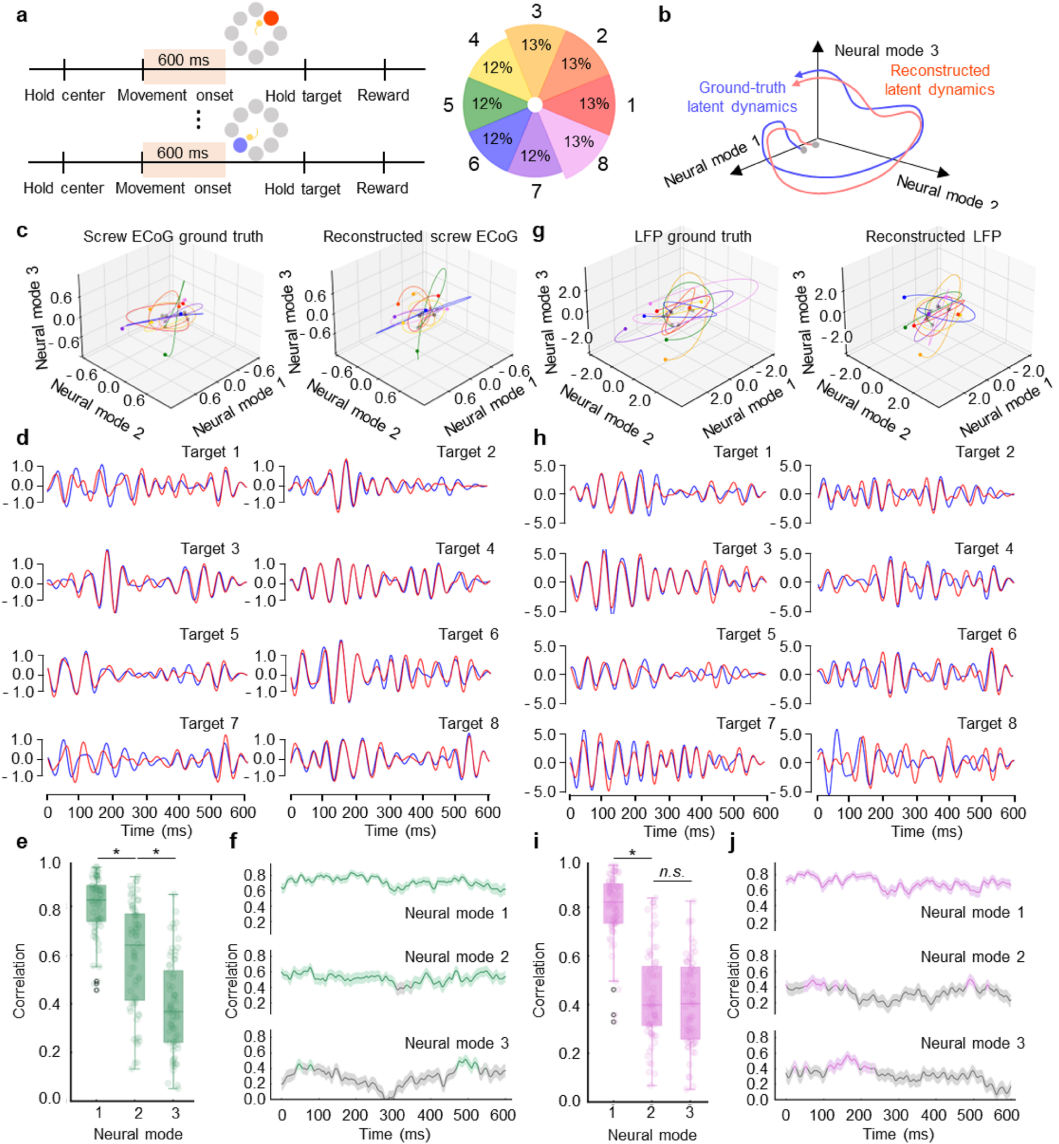
NBGNet captures and reconstructs the latent dynamics in the reaching-out task. (**a**) Schematic of protocol indicates the time window used for analysis. Probability of each target direction is uniform. (**b**) We predicted that the latent dynamics can be recovered. (**c**) Representative latent trajectories derived from the ground-truth screw ECoG (left) and reconstructed screw ECoG (right). Each color represents each target direction in (**a**). (**d**) Projection of average ground truth (blue trace) and reconstructed (red trace) latent trajectories for each target on the first mode. (**e**) Bar plot showing the strong magnitude of the correlations between the ground truth and reconstructed latent trajectories (error bars, s.e.m.; n = 68). (**f**) Temporal correlation trajectories for each neural mode (green trace when above the threshold as 0.4; grey trace as below the threshold; mean ± s.e.m.). (**g**) Same as (**c**) for the inverse model to reconstruct the latent trajectories derived from LFPs. (**h**) Same as (**d**) for the projection of average ground truth LFPs-derived (blue trace) and reconstructed LFPs-derived (red trace) latent trajectories. (**i**) Same as (**e**) for the correlation between the latent trajectories obtained from recorded LFPs and estimated LFPs. (**j**) Same as (**f**) for the inverse model (purple trace when above the threshold as 0.4; grey trace as below the threshold). *p < 0.05 using two-sided Wilcoxon’s rank-sum test. *n.s.* indicates no significant difference.

Since low-dimensional latent dynamics have been widely used to illuminate the relationship between neural population activity and behavior^37–41^, we also test whether NBGNet captures latent dynamics. The window of interest starting from movement onset and ending 600 ms after movement onset is selected, and there is no issue of imbalance target directions (Fig. 5a). We compute the neural manifold and the latent dynamics within it using principal component analysis (PCA)^42,43^. The resulting PCs are termed the neural modes. The first three neural modes capture the majority of the variance, and are used to define the axes of the neural manifold. We then perform canonical correlation analysis (CCA) ^44–46^ to align the latent dynamics (Fig. 5b). Correlation analysis (Pearson’s *ρ*) is utilized to quantify the similarity between these latent dynamics. Since canonical correlations are sorted from the largest to the smallest, we expect the same trend in the evaluation. First, we show that the single-trial and session-average latent trajectories of ground truth and reconstructed screw ECoG are similar for all the target directions (Fig. 5c,d). Strong and a moderate correlation are obtained for neural mode 1 (0.80) and mode 2 (0.60), respectively (Fig. 5e). To assess the effects of behavioral states on the model performance, we calculate the instantaneous correlation across time for each trial. We demonstrate both mode 1 and 2 exhibit a consistently strong correlation (Fig. 5f). These observations hold for the inverse-NBGNet, where latent trajectories derived from the inferred and the ground-truth LFPs are highly correlated (Fig. 5g). Similarly, session-averaged latent traces for the first neural mode are almost the same for all the targets (Fig. 5h). A strong correlation, as well as correlated instantaneous correlation, are also observed in neural mode 1 (0.69; Fig. 5i,j). Neural mode 2 exhibits a relatively poorer performance due to less precise inference from inverse-NBGNet. The results indicate that the NBGNet captures the latent dynamics. As expected, a stronger correlation is associated with the higher ratio of variance that the neural mode explained.

### Performance of linear decoder with NBGNet estimations

**Figure 6:**
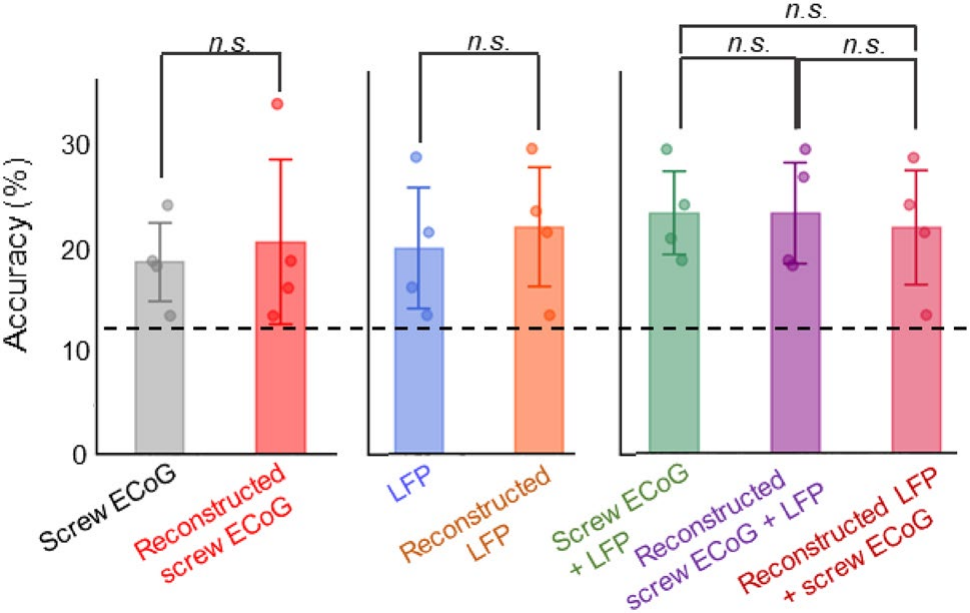
NBGNet inference can be used to predict the movement behavior. Bar plot showing the classification accuracy for each dataset (dashed line, chance performance; error bars, s.d.; n = 4). *p < 0.05 using two-sided paired T-test. *n.s.* indicates no significant difference.

To understand the information encoded within neural populations, decoding cortical activity is of particular interest^45^. We wondered how accurately linear decoders trained with the model-inferred neural activities would perform. We first extract candidate features from the dataset and picked fourteen of them using Fisher score^47^, where fourteen features yields the highest classification accuracy via grid search. Linear discriminant analysis (LDA)^47^ classifiers are then trained with the selected features to predict the direction of cursor’s movement. The classification accuracy is evaluated using fourfold cross-validation. Candidate features are arranged in descending order based on Fisher score averaging across all the channels. LDAs are trained with seven conditions: (1) screw ECoG only, (2) reconstructed screw ECoG only, (3) LFP only, (4) reconstructed LFP only, (5) screw ECoG + LFP, (6) reconstructed screw ECoG + LFP, and (7) screw ECoG + reconstructed LFP. Fourteen features are selected for classifiers 1–4; while twenty-eight features (two-fold increase due to more candidate features available from two datasets) are selected for classifiers 5–7. We demonstrate that no significant difference in classification accuracy between the model inference and the ground truth is observed (p > 0.05; Fig. 6), indicating that NBGNet’s inference maintains the discriminant power. As expected, the classifier trained with LFP and screw ECoG outperforms the other conditions. Surprisingly, the classifiers trained with both real signals and with the inclusion of NBGNet’s predictions (reconstructed screw ECoG + LFP and screw ECoG + reconstructed LFP) yield a comparable decoding capability. Together, we show that the presented model maintained the integrity of information represented by the neural activity.

### Stable performance across days

**Figure 7:**
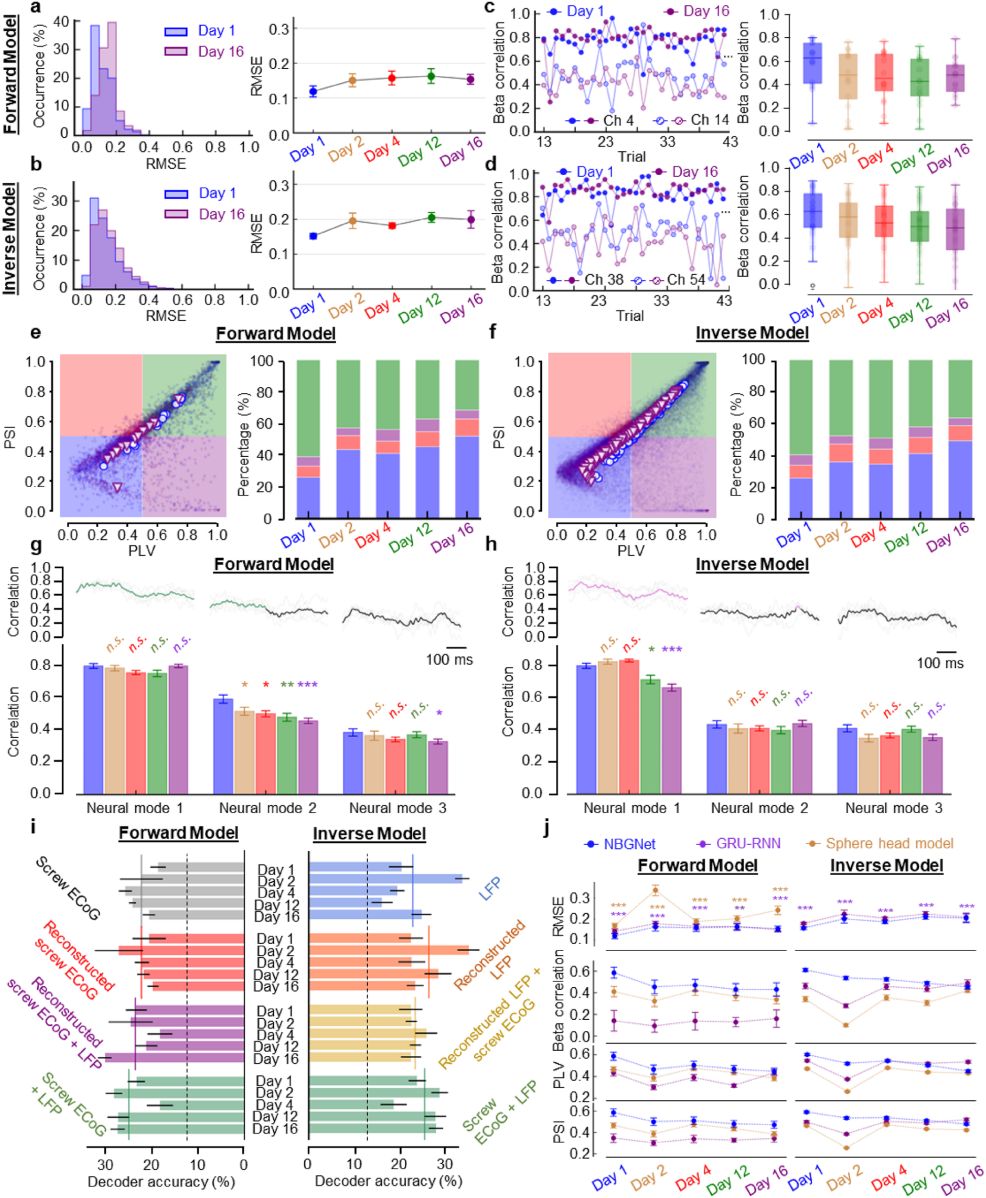
Stability of NBGNet’s predictions for multiple days. (**a**,**b**) Histogram of RMSE (left) at Day 1 (blue) and 16 (purple) for the forward (**a**) and the inverse (**b**) model. Scatter plot of average RMSE (right) showing no significant difference (error bars, s.e.m.; n = 16 and 157 for (**a**) and (**b**); *p* = 0.29 and 0.07 for a and b using one-way ANOVA test). (**c**,**d**) Beta correlation (left) at Day 1 (blue) and 16 (purple) for the forward (**c**) and the inverse (**d**) model. Scatter plot of average beta correlation, where black solid line is obtained by averaging over the channels and black dashed line left the poorest channel out (error bars, s.e.m.; n = 7 and 16 for (**c**) and (**d**)). *p = *0.32 and < 0.05 for **c** and **d** using one-way ANOVA test. While there are some significant decreases for the inverse model, the effect size is small. (**e**,**f**) Scatter plot of PSI versus PLI (left) at Day 1 (blue circle) and 16 (purple triangle) for the forward (**e**) and the inverse (**f**) model. Stacked bars (right) demonstrate the percentage of predictions locating in each section. (**g**,**h**) Temporal correlation averaging across days (upper), where colored segments represents stronger correlation as compared with the grey counterparts. Bar plot of average correlation (lower) exhibiting stable performance (error bars; s.e.m.; n = 68, 49, 128, 78, 135 at Day 1, 2, 4, 12, 16). *p < 0.05, **p < 0.01, ***p < 0.001 using two-sided Wilcoxon’s rank-sum test. *n.s.* indicates no significant difference. (**i**) Bar plots showing the classification accuracy of linear classifier to predict the target direction (error bars, s.d.; n = 5). Solid line represents the average performance across days. (**j**) Performance comparisons between the NBGNet, GRU-RNN, and the sphere head model. **p < 0.01, ***p < 0.001 using two-sided Wilcoxon’s rank-sum test.

As experiments are often conducted across multiple sessions or days, whether the trained model could generate a reliable and robust result is crucial. Here we examine the stability of NBGNet using the same metrics presented in the previous sections. We would like to emphasize that the NBGNet was trained on day 1 and remains fixed for testing in subsequent days. First, the average RMSE for both forward and inverse models are consistent over weeks to a degree almost indistinguishable from that in Day 1 (Fig. 7a,b). As is the case for RMSE, the beta correlation is stable as well even with a few individual trial exceptions (Fig. 7c,d). Specifically, we find an unexpected decrease in correlation of a specific channel (Day 2 for forward model; Day 16 for inverse model) due to the change of the order of magnitude in the measurements. Overall, the predicted neural activities are still highly correlated with the empirical recordings (ρ = 0.47 and 0.52 for forward and inverse models). We then test the stability in phase analysis. While the performance slightly dropped with time, Forward-NBGNet-inferred screw ECoG signals are still highly synchronized with the real recordings (Fig. 7e). More predictions are mostly categorized in the moderate to strong synchrony zone than in the poor one (+ 48%, + 13%, + 17%, + 9%, − 4% for Day 1, 2, 4, 12, 16, respectively). Similarly, reconstructed LFPs are in sync across sessions (+ 10%/session; Fig. 7f).

The NBGNet maintains the capability of reconstructing latent dynamics during the repeated movement generation for the full length of recordings from the monkey (Fig. 7g,h). The stability holds for a range of manifold dimensionalities from 1 to 3. As we find in Day 1 (Fig. 5e,i), the descending trend in the correlations of neural modes is observed for multiple days. The average temporal correlations also show similar results for both forward and inverse model. We then test whether NBGNet inferences predict behavior in different sessions. It is noted that the classifiers performed as well as that trained in different sessions (Fig. 7i). These results provide evidence that NBGNet-derived signals predict behavioral variables with similar accuracy as compared with the ground-truth signals for multiple sessions.

### Comparison of NBGNet and well-known algorithms

Here we compare the NBGNet with two conventional alternatives, specifically a sphere head model^48,49^ and GRU-RNN. The former represents the purely electrophysiology-based approach and the latter one represents the purely data-driven method. The sphere head model provides analytical formulas describing the contribution from current sources to EEG potentials with the assumption of a multi-layered spherical head where each layer represents each brain tissue. The inverse computation can then be achieved by solving the inverse problem. For the state-of-the-art deep learning technique, we have considered several options (e.g., RNN, regularized RNN, long short-term memory based RNN, and GRU-RNN) and finally chose the GRU-RNN due to its highest performance. Accordingly, we apply GRU-RNN as a purely date-driven alternative. The NBGNet outperforms the purely data-driven GRU-RNN and electrophysiology-based sphere head model for multiple days by the smallest RMSE, greatest beta correlation, PLV, and PSI (Fig. 7j). As expected, the performance of the analytical solution is the poorest due to the non-high-density recordings and the unrealistic assumptions (e.g., isotropic conductivity of the medium). While GRU-RNN clearly performs better than the sphere head model, the NBGNet consistently gives a more accurate inference over multiple days.

## Discussion

The brain consists of a hierarchical system with multiple levels of organization^50^. Growing interest in multi-scale interactions among the genetic, cellular, and macroscale levels has recently inspired a shift from emphasizing neural communication in individual scales to exploring the potential associations between scales. However, how these scales are interlinked is still an open question. In this work, the NBGNet addresses an unmet need to capture the implicit relations of multi-scale brain activity. We demonstrate that the neural activity at one scale can be inferred from one another with consistent performance across multiple days without model retraining.

As neuronal coupling among distinct populations can be linear (synchronous) or nonlinear (asynchronous)^51^, a powerful tool capable of capturing nonlinear interactions is imperative. The NBGNet addresses the two critical issues in studying multi-scale brain networks: characterization and approximation. Either inappropriate characterization or improper approximation can lead to erroneous inferences. Here we utilize a BG approach to derive the nonlinear system dynamics in multi-scale brain network (characterization issue) and employ a deep learning technique to approximate the nonlinear mapping (approximation issue). The BG method enables the integration of multi-domain physical systems by specifying the transfer of energy between system components. To the best of our knowledge, this is the first time that the BG is applied to the brain. We model the transfer of electrical energies among brain tissues, but these energies can be measured at different scales or by different approaches. Inherited from BG modeling, another important feature of the NBGNet is system identification^23,52,53^. After network training, the system parameters are extracted and further utilized to interpret the temporal evolution of the underlying dynamical system. The embedded dynamics in the NBGNet are thus able to illustrate how the activity at one scale communicates with other scales, serving as key factors in uncovering the mechanistic understanding of brain computations and the mediation of the behaviors. Although this work demonstrates the power of NBGNet in offline analysis, it could also be implemented online after fitting parameters with a training dataset.

The guiding factor in model evaluation is utilizing comprehensive metrics. This is especially important for neuroscientific research. A perfect performance in one metric may not guarantee the same observation in another. RMSE (Figs. 1, 6) is used to indicate the absolute measure of fit. For similarity analysis in time- and phase-domain, we assess cross-correlation (Fig. 2) and phase synchrony (Fig. 3) between model predictions and ground truths. As a key to understanding neural mechanisms, the capability of reconstructing the low-dimensional latent dynamics is also examined (Fig. 5). Note that both the ground-truth and the reconstructed latent trajectories projected onto the first two jPCs derived by jPCA exhibit rotational dynamics (Supplementary Fig. 2). Additionally, we consider the decoding accuracy as an indicator of the applicability to brain-machine interface (BMI) paradigms (Fig. 6). Despite the suboptimal decoder performance, the NBGNet estimations are shown to decode the cortical activity with similar accuracy. Since there is no evidence indicating the poor trajectories to be excluded from the analysis (Supplementary Fig. 3), exploration of more candidate features and consideration of nonlinear decoders hold great potential to optimize the decoder capability. Both the applications of latent dynamics reconstruction and BMI decoder benefit from the capability of cross-scale modeling. The success of modeling cross-scale effects leads to accurate reconstructions that capture ground-truth latent dynamics and thus provides supplementary information to improve the decoding capability by 1.18-fold. Finally, the predictive power without retraining the model over a long period has recently drawn growing attention in the field of neural engineering. We validate the NBGNet as a reliable approach with the aforementioned metrics and show its broad applicability (Fig. 7).

As the model performance with limited channels is of particular interest, we randomly chose 16 LFP channels and 7 screw ECoG channels from distinct areas to obtain a subset of anatomically spatially distributed signals. It is worth noting that the NBGNet still yields a similar performance if only a subset of measurements is accessible. Interestingly, NBGNet’s performance is not dependent upon the depth, but on the regions (Fig. 2). Forward-NBGNet captures the internal dynamics for performing a center-out task and thus accurately reconstructs the task-related neural activity in premotor, prefrontal cortex, and primary motor cortex. Furthermore, inverse-NBGNet-inferred activity matches the ground truth not only at the cortical region but also at a deeper subcortical area. As the inverse model is developed by nonlinearizing the inversion of linear forward mapping rather than the direct inversion of nonlinear forward mapping, a slightly poorer performance is expected in more ventral brain regions. Additionally, evidenced with the failure of capturing the noise from unstable recordings, dynamics embedded in the NBGNet are useful for disambiguating brain computations.

The bias-variance trade-off is a critical problem in statistics and machine learning^54^, where the simple models have a lower variance yet a higher bias, and the complexity of the model can reduce the bias but increase the variance. It is thus expected that the NBGNet outperforms the analytical sphere head model and the GRU-RNN. With the assumptions of the dipole as the signal sources and the conductivity of the brain tissues, the sphere head model provides a simple solution but leads to a large bias error. The data-driven GRU-RNN enables the approximation of nonlinear dynamics; however, a large variance, or the so-called “overfitting,” can be observed. Therefore, to make a fair comparison, we train the GRU-RNN with appropriate regularization. However, the regularized GRU-RNN is still a black box without any physiological interpretation of the model. Combining both neurobiological modeling and deep learning techniques, the NBGNet succeeds in capturing the patterns in the training data and adapting itself to unseen data. With the complexity lying between sphere head model and GRU-RNN, the NBGNet holds great potential to resolving the bias-variance dilemma.

The NBGNet is powerful for investigating the underlying dynamics in multi-scale brain networks. Modeling the neural activity at disparate scales yields causal interactions among multiple levels, which is crucial in illuminating the mechanistic understanding of brain computation. Effective connectivity extracted from the NBGNet exhibits both unique and shared patterns of both visual feedback and voluntary movement, suggesting that the NBGNet serves as a useful tool to study brain computation. Whereas current work focuses on cross-scale interaction, within-scale communication can be incorporated for comprehensive modeling. Additionally, NBGNet can potentially improve the applicability of brain-machine interfaces by inferring the brain activity with increased signal-to-noise ratio and even combining multi-scale activity^55^. Moreover, the inverse computation to reconstruct the activity at the uncovered brain regions makes LFP-derived whole-brain dynamics available. We are also continuing to explore the potentials of these latent state variables and modeled electrical components in the NBGNet (e.g., whether these components can be utilized for latent dynamics estimations and BMI decoder more effectively). Taken together, our work represents an important step forward towards the mechanistic modeling of multi-scale neural activity, which may facilitate our understanding of neuropathological activity and the development of clinical devices and rehabilitative therapies to treat abnormal neural activity underlying dysfunctional behaviors.

## Methods

### The NBGNet model

#### The NeuroBondGraph network

To introduce the NBGNet (Fig. 1), we start with a generic dynamical system, where the evolution of latent variables and the output is described by nonlinear functions of latent states and the corresponding input. The system dynamics are derived from the BG^22^, modeling the translation between two recording modalities (Supplementary Fig. 1).

#### Bond graphs modeling

BG is a graphical representation of a physical system that allows easy access to the state-space representation. BG consists of the bonds and the elements (Fig. 1a). The bonds represent the power, and each of them has two features: half-arrow and causality. The power is broken down into two pairs: flow (e.g., current in electrical domain) and effort (e.g., voltage). Half-arrow indicates the sign convention for the work being done. Accordingly, sources will always have the arrow pointing away from the element, while others will have the arrow pointing into the elements. Causality in BG denotes which side of the bond governs the instantaneous power. There are multiple categories for elements, including (1) sources, denoted as **S**, serving as the input to the system, (2) sinks, denoted as **S** as well while serving as the output of the system, (3) inertia elements (e.g., inductance), denoted as **I**, which store energy, (4) resistance elements (e.g., resistance), denoted as **R**, which dissipate energy, (5) compliance elements (e.g., capacitance), denoted as **C**, which store potential energy, and (6) 0- or 1-junctions which split the power. Specifically, 0-junctions are that all efforts are equal across the bonds and the sum of flow in equals to the sum of flow out. In contrast, 1-junctions represent that all flows are equal across the bonds and the sum of effort in equals to the sum of effort out. Furthermore, as two passive components, **I** and **C**, exhibit time-dependence behavior, there exists preferred causal orientations with **C** defining the effort and **I** defining the flow. Since energy in different domains can be transferred into each other with a constant, BGs enable modeling of physical systems in distinct domains.

#### Bond graphs forward and inverse modelling

The interactions between measurements are modeled based on the physiology of brain tissue and its effect on the electrical signal flow. In this work, screw ECoG signals are recorded within the skull while LFP signals are measured within the cortical and subcortical structures (Fig. 1c,d). Therefore, the biological medium between the recording locations consists of skull, dura mater, and/or cortex. We then model the signal translations as an electrical circuit with the LFP as the source, the brain tissues as effective impedance, and the screw ECoG as the voltage measurement (Supplementary Fig. 1a). Since the skull contains sinus cavities and numerous foramina, a three-layer structure is utilized: a spongy bone layer in the middle of two compact bone layers. The cavities in the spongy bone are modeled as a capacitance that provided potentials inside them. In contrast, the compact bone and the trabeculae of the spongy bone are modeled as resistances. All potential paths for electrical signals to travel are considered to model the signal propagation. As a thick membrane surrounding the brain, dura mater is represented with the effective resistance and capacitance in parallel. Although the cortex is composed of folded grey matter, we model it as an effective resistance to simplify the complexity. Combining the modeling of impedances of brain tissues together, the LFP-screw ECoG transmission electrical circuit is established (Supplementary Fig. 1a), followed by the generation of the BG (Supplementary Fig. 1b). The compliance components ***C*** indicate the hidden state variables in the dynamic equations. Ultimately, we obtain a 3rd order ordinary differential equation describing the dynamics underlying multi-scale system based on the constitutive equation for each element and connection (Supplementary Note 1).

The multi-variable time-varying BG forward model is expressed using a state-space representation (Supplementary Note 1). The inverse of the forward model is then obtained by an inversion algorithm^56^. Similarly, another 3rd order ordinary differential equation is derived associated with the inverse model (Supplementary Note 2). Eventually, both the forward and inverse models are expressed as the following state-space representation,

$$\dot{x}=\mathbf{A}x+\mathbf{B}u$$

$$y=\mathbf{C}x+\mathbf{D}u$$

where, $x$ represents the hidden variables, $u$ is the input vector, $y$ is the output vector, and $\mathbf{A},\mathbf{B},\mathbf{C},\mathbf{D}$ are the system-dependent matrices (Supplementary Notes 1, 2).

#### The full NBGNet inference model

Here, we develop the deep learning technique termed NBGNet to approximate the unknown nonlinear relationship. In NBGNet, the network implements the causal form of the dynamic equations where the unknown nonlinear mappings are realized by the MLP units (Supplementary Fig. 1c,d). The RNN framework is then utilized to capture the cross-scale interactions by maximizing the likelihood of the observed brain signals with its internal states. The modeling of brain tissue impedances makes NBGNets neurobiologically realistic to analyze neural signals. Here we demonstrate the capability of extracting bidirectional cross-scale dynamics using NBGNets for forward and inverse models, respectively. The evolution of latent variables and the output is described by the nonlinear functions approximated by NBGNets.

$${{\varvec{V}}}_{screwECoG,t},{{\varvec{q}}}_{t+1}^{forward}=NB{G}^{forward}\left({{\varvec{q}}}_{t}^{forward},{{\varvec{V}}}_{LFP,t}\right),$$

$${{\varvec{V}}}_{LFP,t},{{\varvec{q}}}_{t+1}^{inverse}=NB{G}^{inverse}\left({{\varvec{q}}}_{t}^{inverse},{{\varvec{V}}}_{screwECoG,t}\right),$$

where ***q*** represents the latent states of the system, and ***V*** represents the electrical recordings. The forward-NBGNet serves as a forward solution that models the single-trial screw ECoG as a nonlinear recursive mapping from the multivariate LFP (Fig. 1a,b). The network’s units to approximate such a mapping depend on three elements: a trial-specific initial state, input signals, and the parameters defining the system dynamics. To mimic the real-time modeling and abide by causality constraints, the network only runs through the trial forward for estimation. By inverting the forward solution, the inverse-NBGNet is then developed to predict LFP from screw ECoG (Fig. 1b). As inverse computation is an ill-posed problem which can lead to a non-unique and unstable solution^49^, we expect a relatively poorer performance as compared with the forward solution.

To optimize the NBGNet, we train our model to minimize the mean-squared-error between predicted activity and the ground-truth using simultaneously recorded LFP data from the left hemisphere and screw ECoG data from both hemispheres. The major hyperparameters for forward and inverse model are the number of hidden nodes in the MLP unit for nonlinear mapping estimation and the time step. For both forward and inverse model, 7 nodes are utilized in MLP units, and the time step of NBGNet is equal to the data sampling rate. Over-fitting occurs when we train the model with the same batch of data for excessive iterations. To avoid over-fitting, we select different trials of data for training when NBGNet has been updated for twenty times. The NBGNets are randomly initialized by Glorot uniform initializer and optimized using adaptive moment estimation (Adam) optimizer with a starting learning rate of 1 × 10^–3^. A portion of the data serve as the validation set and to determine if the model was overfit. Here we used a ratio of 9:1 between training and validation (held-out) data. After model training, the parameters of the NBGNet remain fixed for further analysis.

In this work, we analytically validate our NBGNets by yielding small root mean squared errors (Fig. 1g,h); reproducing features commonly seen in neuroscientific analyses (cross-correlation; Fig. 2, phase synchrony; Fig. 3); capturing cross-scale interactions aligning well with the abstraction of the hierarchical anatomy of the mammalian nervous system (Fig. 4); reconstructing low-dimensional latent dynamics (Fig. 5); inferring details of behavior (Fig. 6); and predicting out-of-sample conditions (Fig. 7). For all results in this paper, we train NBGNets without any information about task conditions or behavioral parameters (e.g., real kinematics or eye-tracker data) and present the results from testing data.

### Ethics statement

All the experiments were performed in compliance with the regulation of the Animal Care and Use Committee at the University of California at Berkeley. The study is approved by the ethics Committee of University of California at Berkeley. The subject was approximately 6 years of age at the time of data collection*.*

### Experimental model and subject details

A male rhesus macaque is used in these experiments. The macaque is trained to perform a center-out task (Fig. 1e,f). Briefly, the subject is trained to use a joystick to move a cursor on a computer screen from a center target to a peripheral target. The joystick is attached to the front of the primate chair and the subject is free to use either hand to control the joystick during the experiment. In the task, the subject is trained to hold the cursor at the center target shown on the screen for 320 ms. Then the subject is presented with one of the eight outer targets, equally spaced in a circle, and selected randomly with uniform probability. The subject moves the cursor to the peripheral target and holds the cursor inside the target for 320 ms. A trial is successful if the subject completes the 320 ms center-hold followed by holding at the peripheral target for 320 ms. The reward is scheduled after a successful trial, where a custom-programmed Arduino triggered the reward system to deliver a small amount of juice to the subject.

### Scale-dependent analysis

To evaluate how close the model predictions are to the ground-truth signals, root mean square error (RMSE) is commonly used to indicate the absolute fit of the model. RMSE is defined as the square root of the mean of the square of the error,

$${\text{RMSE}}=\sqrt{\frac{1}{T}\sum_{t=1}^{T}{\left[{\mathbf{Y}}_{gt}\left(t\right)-{\mathbf{Y}}_{pre}(t)\right]}^{2}}$$

where the ${\mathbf{Y}}_{gt}$ represents ground-truth measurement, ${\mathbf{Y}}_{pre}$ represents the model prediction, and $T$ is the number of time points in the given trial.

### Similarity analysis

Similarity of two time series signals also conveys an important message whether two time series signals exhibit similar shape of oscillation. Here we use Pearson correlation coefficient to measure how highly correlated two time series signals are.

$$\uprho \left({\mathbf{Y}}_{gt},{\mathbf{Y}}_{pre}\right)=\frac{\sum {\mathbf{Y}}_{gt}{\mathbf{Y}}_{pre}-\frac{\sum {\mathbf{Y}}_{gt}\sum {\mathbf{Y}}_{pre}}{T}}{\sqrt{\left(\sum {\mathbf{Y}}_{gt}^{2}-\frac{{\left(\sum {\mathbf{Y}}_{gt}\right)}^{2}}{T}\right)\left(\sum {\mathbf{Y}}_{pre}^{2}-\frac{{\left(\sum {\mathbf{Y}}_{pre}\right)}^{2}}{T}\right)}}$$

where the ${\mathbf{Y}}_{gt}$ represents ground-truth measurement, ${\mathbf{Y}}_{pre}$ represents the model prediction, and $T$ is the number of time points in the given trial.

### Phase analysis

Phase-domain reveals other characteristics that are not visible in time-domain. Phase synchronization between neurons is a fundamental neural mechanism that supports neural communication and plasticity^57^. Given a pair of signals, **s**_1_(t) and **s**_2_(t), which have been band-pass filtered to a frequency range of interest, the Hilbert transform, **HT**[⋅],is applied to obtain the corresponding analytical signals, **z**_1_(t) and **z**_2_(t):

$${\mathbf{z}}_{i}\left(t\right)={\mathbf{s}}_{i}\left(t\right)+j \mathbf{H}\mathbf{T}\left[{\mathbf{s}}_{i}(t)\right]={\mathbf{A}}_{i}(t){e}^{j{{\varvec{\upphi}}}_{i}(t)}$$

$$\mathbf{H}\mathbf{T}\left[{\mathbf{s}}_{i}({t}_{k})\right]={\mathbf{s}}_{i}\left({t}_{k}\right)*\frac{1}{2\pi }\left[{\int }_{-\pi }^{0}j\cdot {e}^{jwk}dw-{\int }_{0}^{\pi }j\cdot {e}^{jwk}dw\right]$$

where $k=1$ to $T$, ${\mathbf{A}}_{i}(t)$ represents the instantaneous amplitude, and ${{\varvec{\upphi}}}_{i}(t)$ represents the instantaneous phase. In order to obtain a comprehensive view, we utilized two metrics: phase-locking value and phase synchrony index. Phase locking value^31^, $PLV$, or so-called mean phase coherence^32^, is defined as,

$$PLV=\left|\frac{1}{T}\sum_{i=0}^{T-1}{e}^{j(\Delta {\varvec{\upphi}}(t))}\right|$$

where Δ**ϕ**(*t*) represents the phase difference between pair of signals.

This metric characterizes the intra-trial variability of the phase difference between two signals, where a larger PLV indicates a stronger synchrony between them. In addition, the phase of phase-locking can be extracted to evaluate the mean phase difference across time.

In addition to the PLV, we are also interested in the instantaneous performance, and thus we consider phase synchrony index. First, provided with the instantaneous phase of two time series signals, **ϕ**_1_(t) and **ϕ**_2_(t), the instantaneous phase synchrony (IPS)^58^, which measured the phase similarity at each timepoint, is calculated by

$$IPS(t)=1-{\text{sin}}\left(\frac{\left|{{\varvec{\upphi}}}_{1}\left(t\right)-{{\varvec{\upphi}}}_{2}\left(t\right)\right|}{2}\right)$$

where the phase is in the unit of degree. IPS spans the range of 0–1, where a larger value indicates a stronger synchrony. We define a quarter of the whole range of phase difference (180°), 45°, as the threshold. When the phase difference is less than 45°, IPS was greater than 0.62, thus revealing a better performance. We then calculated the ratio of the time with the IPS greater than 0.62, termed phase synchrony index (PSI; Fig. 3),

$$PSI=\frac{{t}_{IPS>0.62}}{T}$$

To determine the level of the phase synchrony, we categorize the two-dimensional scatter plot of $PSI$ and $PLV$ into four sections with both thresholds as 0.5: Zone 1 (low $PSI$ and low $PLV$) indicates poor synchronization, Zone 2 (low $PSI$ and high $PLV$) indicates medium synchronization, Zone 3 (high $PSI$ and low $PLV$) indicates medium synchronization, and Zone 4 (high $PSI$ and high $PLV$) indicates perfect synchronization (Fig. 3).

### Neural latent dynamic analysis

To characterize the latent dynamics associated with the recorded or reconstructed neural activity in each trial, we analyze the filtered signals, which are obtained by applying a bandpass filter with cutoffs at 12.5 Hz and 30 Hz, in the window starting at movement onset and ending 600 ms after movement onset. Such a window is selected due to the interest in movement execution during the trial. For each trial, we obtain the data matrix *D* of dimension *n* by *T*, where *n* was the number of recorded channels, *T* was the number of time points in the given trial. Then we compute the low-dimensional manifold by applying principal component analysis (PCA)^42^ to *D*. The resulting PCs are the linear combination of measurements of all the channels. We then rank these PCs based on the amount of neural variance explained by each PC. We keep only the three leading PCs to represent the low-dimensional manifold, where these three leading PCs, referred to as neural modes, explain most of the variance in the data matrix.

Differences between the neural recordings and the NBGNet’s predictions necessarily cause a change in the estimated manifold and latent dynamics; however, a simple linear transformation can be applied to compensate for these differences^59^. Here we expect to identify the embedding space where true latent dynamics are located by using canonical correlation analysis (CCA). In CCA, given a pair of two latent trajectories, **P**_*A*_ and **P**_*B*_, linear transformations for each trajectory are identified to make the linearly transformed latent trajectories, ${\widetilde{{\varvec{P}}}}_{A}$ and ${\widetilde{{\varvec{P}}}}_{B}$, maximally correlated. First, QR decomposition^60^ is applied to both latent trajectories,

$${{\varvec{P}}}_{A}^{T}={{\varvec{Q}}}_{A}{{\varvec{R}}}_{A},$$

$${{\varvec{P}}}_{B}^{T}={{\varvec{Q}}}_{B}{{\varvec{R}}}_{B}.$$

Then the singular value decomposition is performed on the inner product of **Q**_*A*_ and **Q**_*B*_*:*

$${{\varvec{Q}}}_{A}^{T}{{\varvec{Q}}}_{B}={\varvec{U}}{\varvec{S}}{{\varvec{V}}}^{T}.$$

The transformation matrix, **T**_*A*_ and **T**_*B*_, is then obtained by:

$${{\varvec{T}}}_{A}={{\varvec{R}}}_{A}^{-1}{\varvec{U}},$$

$${{\varvec{T}}}_{B}={{\varvec{R}}}_{B}^{-1}{\varvec{V}}.$$

Accordingly, the transformed latent trajectories are given by:

$${\widetilde{{\varvec{P}}}}_{A}^{T}={{\varvec{P}}}_{A}^{T}{{\varvec{T}}}_{A},$$

$${\widetilde{{\varvec{P}}}}_{B}^{T}={{\varvec{P}}}_{B}^{T}{{\varvec{T}}}_{B}.$$

The correlation between the transformed latent trajectories, termed canonical correlation (CC), is obtained by the Pearson correlation coefficient. As CC was sorted from the largest to the smallest in CCA, we expect to observe a descending order from neural mode 1 to mode 3.

### Features selection for decoding the direction of the movement

We consider several features per channel as candidates for the decoder and select the leading number of features for further analysis. For each channel, we obtain a total of 34 features, including root mean square (RMS), mean frequency (MF), waveform length (WL), and the power at certain frequency ranged from 10 to 40 Hz (step size as 1 Hz):

$$RMS=\sqrt{\frac{1}{T} \sum \limits_{t=0}^{T-1}{\varvec{Y}}{\left(t\right)}^{2}},$$

$$MF=\frac{{\sum }_{t=0}^{T-1}{f}_{t}{p}_{t}}{{\sum }_{t=0}^{T-1}{p}_{t}},$$

$$WL =\sum \limits_{t=1}^{T-1}| {\varvec{Y}} (t)-{\varvec{Y}}(t-1)|,$$

where **Y**(t) represents the neural signals, T is the number of time points in the given trial, and f_t_ and p_t_ are the frequencies of the power spectrum and the corresponding amplitude.

To determine the subset of features selected for decoders, we calculate the Fisher score^61^ for each candidate feature. The Fisher score, F(x^i^), for the i-th feature, x^i^, is computed by

$$F\left({x}^{i}\right)=\frac{{\sum }_{j=1}^{c}{n}_{j}{\left({\mu }_{j}^{i}-{\mu }^{i}\right)}^{2}}{{\sum }_{j=1}^{c}{n}_{j}{\left({\sigma }_{j}^{i}\right)}^{2}},$$

where μ^i^_j_ and σ^i^_j_ are the mean and standard deviation of the j-th class corresponding to the i-th feature, μ^i^ denotes the mean of the whole data set corresponding to the i-th feature, n_j_ represents the size of the j-th class, and c is the total number of classes. After computing the Fisher score for each feature, we select the top fourteen ranked features to predict the subject’s behavior. Number of features is determined by maximizing the classification accuracy via grid search.

To test whether the reconstructed activity from the NBGNet maintain movement-related information, we build linear decoders to predict the direction of the movement based on the neural activity. Our hypothesis is that our NBGNet inference and the neural recordings will yield a comparable classification accuracy. To test this hypothesis, we compare the predictive accuracy of seven types of decoders: (1) a decoder trained and tested based on screw ECoG; (2) a decoder trained and tested based on reconstructed screw ECoG inferred by forward-NBGNet; (3) a decoder trained and tested based on LFP; (4) a decoder trained and tested based on reconstructed LFP inferred by inverse-NBGNet; (5) a decoder trained and tested based on screw ECoG and LFP; (6) a decoder trained and tested based on reconstructed screw ECoG and LFP; and (7) a decoder trained and tested based on reconstructed LFP and screw ECoG. All decoders are defined using linear discriminant analysis with the selected features as inputs to predict the direction of cursor’s movement. They are trained and tested on the same day, using a fourfold cross-validation procedure to protect against overfitting. Chance-level performance is obtained by shuffling the dataset. As expected, all predictive accuracy is higher than chance-level (~ 12.5%).

### Comparison methods

The multi-scale modeling is relatively new in the neuroscience field. To benchmark performance of NBGNet relative to other existing algorithms, we implement two approaches: the electrophysiology-based sphere head model^15^ and data-driven RNN^62^. The sphere head model is widely used to either compute the contribution from the current dipoles to the electrical potentials recorded at scalp electroencephalography (EEG) or estimate the current dipole sources based on the scalp potentials^63^. Typically, the sphere head model assumes the head to be modeled as a four-layered sphere (brain, cerebrospinal fluid, skull, and scalp). Using the quasi-static approximation of Maxwell’s equations and the volume-conductor theory, the electrical potential, **Φ**(**r**,*t*), is obtained by the following Poisson equation^64^:

$$\nabla \cdot \sigma \left({\varvec{r}}\right)\nabla \boldsymbol{\Phi }\left({\varvec{r}}, t\right)=-C({\varvec{r}},t)$$

where *σ*(**r**) represents the position-dependent conductivity of the medium, and *C*(**r**,*t*) is the density of the current sources. Assuming the conductivity to be isotropic, the boundary conditions to the sphere head model were

$${\boldsymbol{\Phi }}^{s+1}\left({{\varvec{r}}}_{s}, t\right)={\boldsymbol{\Phi }}^{s}\left({{\varvec{r}}}_{s}, t\right),$$

$${\sigma }_{s+1}\frac{\partial {\boldsymbol{\Phi }}^{s+1}\left({{\varvec{r}}}_{s}, t\right)}{\partial r}={\sigma }_{s}\frac{\partial {\boldsymbol{\Phi }}^{s}\left({{\varvec{r}}}_{s}, t\right)}{\partial r},$$

$$\frac{\partial {\boldsymbol{\Phi }}^{4}\left({{\varvec{r}}}_{4}, t\right)}{\partial r}=0,$$

where each layer is labelled by *s* = 1 to 4. Here we assume the dipole is radial with magnitude *p(t)* at location *r*_*z*_. The analytical solution is then given by:

$${\boldsymbol{\Phi }}^{1}\left(r, \theta , t \right)=\frac{p(t)}{4\pi {\sigma }_{1}{r}_{z}}\sum \limits_{n=1}^{\infty }\left[{A}_{n}^{1}{\left(\frac{r}{{r}_{1}}\right)}^{n}+{\left(\frac{{r}_{z}}{r}\right)}^{n+1}\right]n{P}_{n}\left({\cos}\left(\theta \right)\right) {r}_{z}<r\le {r}_{1}$$

$${\boldsymbol{\Phi }}^{s}\left(r,\theta , t \right)=\frac{p(t)}{4\pi {\sigma }_{1}{r}_{z}}\sum \limits_{n=1}^{\infty }\left[{A}_{n}^{s}{\left(\frac{r}{{r}_{s}}\right)}^{n}+{B}_{n}^{s}{\left(\frac{{r}_{s}}{r}\right)}^{n+1}\right]n{P}_{n}\left({\cos}\left(\theta \right)\right) {r}_{s-1}\le r\le {r}_{s}$$

where **Φ**^*s*^(*r*,*θ*,*t*) is the extracellular potential measured at radius *r* and the angle *θ* between the measurement and dipole location vectors in the shell *s*, *r*_*s*_ represent the radius of sphere *s*, *A*^*s*^_*n*_ and *B*^*s*^_*n*_ are the coefficients depending on the radius and conductivities of each medium (defined in^15^), and *P*_*n*_(cos(*θ*)) represents the *n*-th Legendre Polynomial. As the solution is implemented into the case where we had *n*_*d*_ current dipoles and *n*_*r*_ recording units, a linear transformation matrix *F* of dimension *n*_*r*_ by *n*_*d*_ is obtained and utilized to convert the dipole moment vectors **X** into the electrical potential **Y**, given by **Y** = *F***X**. This is a so-called forward mapping. When we perform inverse mapping to estimate **X** from **Y**, we need to solve an underdetermined system with pseudo-inverse by minimizing the following equation,

$${\Vert {\varvec{X}}\Vert }^{2}+\lambda \left({\varvec{Y}}-F{\varvec{X}}\right).$$

The solution to minimizing the above equation is given by,

$${\varvec{X}}={F}^{T}{\left(F{F}^{T}\right)}^{-1}{\varvec{Y}}.$$

Here we segment the brain (*n*_*d*_ = 3600), where each segment includes a potential current dipole source. Since our data for comparison does not include dipole sources, we adapt the algorithms into two-step computation for both the forward and inverse models. In the forward model, we perform inverse mapping from LFP toward estimated dipole sources,

followed by a forward mapping from the estimated dipole sources toward screw ECoG recordings. Similarly, in the inverse model, we perform inverse mapping from screw ECoG toward estimated dipole sources followed by a forward mapping from the estimated dipole sources toward LFP recordings. The parameters are summarized in Table 1.

**Table 1:** Parameters for sphere head model.

Label	Tissue	Radius (mm)	σ (S/m)
1	Brain	27.88	0.33
2	Cerebrospinal fluid	28.24	1.65
3	Skull	30.00	0.00825
4	Scalp	31.76	0.33

RNN is a deep learning method widely used to model a nonlinear dynamical system that included nonlinearity, recurrent connection, and hidden dynamic states^65,66^. In order to handle the long-term dependency embedded in the neural activity, Gated recurrent unit (GRU)^62^ is often implemented, where in each time point, model can determine the information to be stored and filtered. GRU is chosen over long short-term memory (LSTM) by its speed and the simpler structure. GRU-based RNN utilized in this work for comparison consists of two GRU layers with 64 and 32 units, two hidden layers with 32 and 16 nodes for forward model or 32 and 128 nodes for inverse model, and the output layer. To avoid overfitting, we train the GRU-RNN with L2 regularization^67,68^ and dropout^69^. The relevant hyperparameters were optimized via Bayesian optimization. The training details, including training iteration, the split ratio of training and validation data, and the choice of optimizer, are set to be the same as NBGNet to ensure a fair comparison.

### Supplementary Information

Supplementary Information.

## Data Availability

All neural data in this study are available from the corresponding author upon reasonable request.

## Code availability

Python scripts for the model will be made available on GitHub.
