# Supplementary material for: Neurobiologically realistic neural network enables cross-scale modeling of neural dynamics
Source: Sci Rep. 2024 Mar 1;14:5145. doi: 10.1038/s41598-024-54593-w (PMC10907713; doi:10.1038/s41598-024-54593-w)
Supplement: Supplementary file 1 — Supplementary Information. [file 41598_2024_54593_MOESM1_ESM.docx]

**Supplementary Information**

**Neurobiologically realistic neural network enables cross-scale modeling of neural dynamics**

**Yin-Jui Chang^1^, Yuan-I Chen^1^, Hsin-Chih Yeh^1,2^, Samantha R. Santacruz^1,3,4^**

*^1^Biomedical Engineering, University of Texas at Austin, Austin, TX, USA*

*^2^Texas Materials Institute, University of Texas at Austin, Austin, TX, USA*

*^3^Institute for Neuroscience, University of Texas at Austin, Austin, TX, USA*

*^4^Electrical and Computer Engineering, University of Texas at Austin, Austin, TX, USA*

**Supplementary Notes:**

Supplementary Note 1: Bond Graph forward modeling

Supplementary Note 2: Bond Graph inverse modeling

**Supplementary Figures:**

Supplementary Figure 1: Development of the NBGNet

Supplementary Figure 2: NBGNet captures and reconstructs rotational latent dynamics in the reaching-out task

Supplementary Figure 3: The classification accuracy with respect to each target direction

**Supplementary Note**

**Supplementary Note 1. Bond Graph forward model.** Combing together, LFP-screw ECoG transmission electrical circuit can be established (**Supplementary Figure 1a**). With the effective electrical circuit for electrical signal pathways, Bond Graph is then generated (**Supplementary Figure 1b**), where the charges, *q*, are represented the states of the physical systems. Given the causality assignment, the system is 3^rd^ order. The ordinary differential equations for the system are derived as follows:

$\begin{matrix} \dot{q}_{1}=\dot{q}_{2}+\frac{q_{2}}{R_{2}C_{2}}+\frac{1}{R_{5}}\left( \frac{q_{2}}{C_{2}}-\frac{q_{3}}{C_{3}} \right)-\frac{q_{1}}{R_{1}C_{1}} \\ \dot{q}_{2}=\frac{1}{1+\frac{R_{0}+R_{screwECoG}}{R_{4}}}\left( \frac{1}{R_{4}}\left( V_{LFP}-\frac{q_{1}}{C_{1}}-\frac{q_{2}}{C_{2}}-\left( R_{0}+R_{screwECoG} \right)\left( \frac{q_{2}}{R_{2}C_{2}}+\frac{1}{R_{5}}\left( \frac{q_{2}}{C_{2}}-\frac{q_{3}}{C_{3}} \right) \right) \right)-\frac{q_{2}}{R_{2}C_{2}}-\frac{1}{R_{5}}\left( \frac{q_{2}}{C_{2}}-\frac{q_{3}}{C_{3}} \right) \right) \\ \dot{q}_{3}=\frac{1}{R_{5}}\left( \frac{q_{2}}{C_{2}}-\frac{q_{3}}{C_{3}} \right)-\frac{q_{3}}{R_{3}C_{3}} \end{matrix}$ [1]

$V_{screwECoG}=R_{screwECoG}\left( \frac{1}{R_{0}+R_{4}+R_{screwECoG}}\left( V_{LFP}-\frac{q_{1}}{C_{1}}-\frac{q_{2}}{C_{2}} \right) \right)$ [2]

While the equations above represent the ideal condition where the resistance *R* and the capacitance *C* are linear. Considering the uncertainty and continuous changing of human’s brain tissue, nonlinearity is introduced in the equation:

$\begin{matrix} \dot{q}_{1}=F_{R_{T}}^{-1}\left( u-F_{C_{1}}^{-1}\left( q_{1} \right)-F_{C_{2}}^{-1}\left( q_{2} \right) \right)-F_{R_{1}}^{-1}\left( F_{C_{1}}^{-1}\left( q_{1} \right) \right) \\ \dot{q}_{2}=F_{R_{T}}^{-1}\left( u-F_{C_{1}}^{-1}\left( q_{1} \right)-F_{C_{2}}^{-1}\left( q_{2} \right) \right)-F_{R_{2}}^{-1}\left( F_{C_{2}}^{-1}\left( q_{2} \right) \right)-F_{R_{5}}^{-1}\left( F_{C_{2}}^{-1}\left( q_{2} \right)-F_{C_{3}}^{-1}\left( q_{3} \right) \right) \\ \dot{q}_{3}=F_{R_{5}}^{-1}\left( F_{C_{2}}^{-1}\left( q_{2} \right)-F_{C_{3}}^{-1}\left( q_{3} \right) \right)-F_{R_{3}}^{-1}\left( F_{C_{3}}^{-1}\left( q_{3} \right) \right) \end{matrix}$ [3]

$y=F_{R_{screwECoG}}\left( F_{R_{T}}^{-1}\left( u-F_{C_{1}}^{-1}\left( q_{1} \right)-F_{C_{2}}^{-1}\left( q_{2} \right) \right) \right)$ [4]

where, $R_{T}$ represents $R_{0}+R_{4}+R_{screwECoG}$, $u$ represents $V_{LFP}$, $y$ represents $V_{screwECoG}$, and $F(\cdot)$ is a nonlinear function to be determined.

**Supplementary Note 2. Bond Graph inverse model.** The multi-variable time varying Bond Graph forward model, Equation 1-2, can be expressed as the state-space representation,

$\dot{x}=\mathbf{A}x+\mathbf{B}u$ [5]

$y=\mathbf{C}x+\mathbf{D}u$ [6]

where, $x=\left[ q_{1}, q_{2}, q_{3} \right]^{T}$, $u=V_{LFP}$, $y=V_{screwECoG}$,

$\mathbf{A}=\left[ \begin{matrix} \left( \frac{-1}{R_{screwECoG}+R_{0}+R_{4}}+\frac{-1}{R_{1}} \right)\frac{1}{C_{1}} & \left( \frac{-1}{R_{screwECoG}+R_{0}+R_{4}} \right)\frac{1}{C_{2}} & 0 \\ \left( \frac{-1}{R_{screwECoG}+R_{0}+R_{4}} \right)\frac{1}{C_{1}} & \left( \frac{-1}{R_{screwECoG}+R_{0}+R_{4}}+\frac{-1}{R_{2}}+\frac{-1}{R_{5}} \right)\frac{1}{C_{2}} & \frac{1}{R_{5}C_{3}} \\ 0 & \frac{1}{R_{5}C_{2}} & \left( \frac{-1}{R_{5}}+\frac{-1}{R_{3}} \right)\frac{1}{C_{3}} \end{matrix} \right]$ [7]

$\mathbf{B}=\left[ \frac{1}{R_{screwECoG}+R_{0}+R_{4}} \frac{1}{R_{screwECoG}+R_{0}+R_{4}} 0 \right]^{T}$ [8]

$\mathbf{C}=\left[ \frac{-R_{screwECoG}}{\left( R_{screwECoG}+R_{0}+R_{4} \right)C_{1}} \frac{-R_{screwECoG}}{\left( R_{screwECoG}+R_{0}+R_{4} \right)C_{2}} 0 \right]$ [9]

$\mathbf{D}=\left[ \frac{R_{screwECoG}}{R_{screwECoG}+R_{0}+R_{4}} \right]$ [10]

The inversion algorithm for multi-variable system were obtained by the following,

$\begin{matrix} \dot{q}_{1}=-\frac{q_{1}}{R_{1}C_{1}}+\frac{V_{screwECoG}}{R_{screwECoG}} \\ \dot{q}_{2}=-\frac{q_{2}}{R_{2}C_{2}}-\frac{1}{R_{5}}\left( \frac{q_{2}}{C_{2}}-\frac{q_{3}}{C_{3}} \right)+\frac{V_{screwECoG}}{R_{screwECoG}} \\ \dot{q}_{3}=\frac{1}{R_{5}}\left( \frac{q_{2}}{C_{2}}-\frac{q_{3}}{C_{3}} \right)-\frac{q_{3}}{R_{3}C_{3}} \end{matrix}$ [11]

$V_{LFP}=\frac{q_{1}}{C_{1}}+\frac{q_{2}}{C_{2}}+\frac{R_{screwECoG}+R_{0}+R_{4}}{R_{screwECoG}} V_{screwECoG}$ [12]

As forward model, nonlinearity is introduced in the equation as well,

$\begin{matrix} \dot{q}_{1}=F_{R_{screwECoG}}^{-1}\left( y \right)-F_{R_{1}}^{-1}\left( F_{C_{1}}^{-1}\left( q_{1} \right) \right) \\ \dot{q}_{2}=F_{R_{screwECoG}}^{-1}\left( y \right)-F_{R_{2}}^{-1}\left( F_{C_{2}}^{-1}\left( q_{2} \right) \right)-F_{R_{5}}^{-1}\left( F_{C_{2}}^{-1}\left( q_{2} \right)-F_{C_{3}}^{-1}\left( q_{3} \right) \right) \\ \dot{q}_{3}=F_{R_{5}}^{-1}\left( F_{C_{2}}^{-1}\left( q_{2} \right)-F_{C_{3}}^{-1}\left( q_{3} \right) \right)-F_{R_{3}}^{-1}\left( F_{C_{3}}^{-1}\left( q_{3} \right) \right) \end{matrix}$ [13]

$u=F_{C_{1}}^{-1}\left( q_{1} \right)+F_{C_{2}}^{-1}\left( q_{2} \right)+F_{R_{T}}\left( F_{R_{screwECoG}}^{-1}\left( y \right) \right)$ [14]

where, $R_{T}$ represents $R_{0}+R_{4}+R_{screwECoG}$, $u$ represents $V_{LFP}$, $y$ represents $V_{screwECoG}$, and $F(\cdot)$ is a nonlinear function to be determined.

**
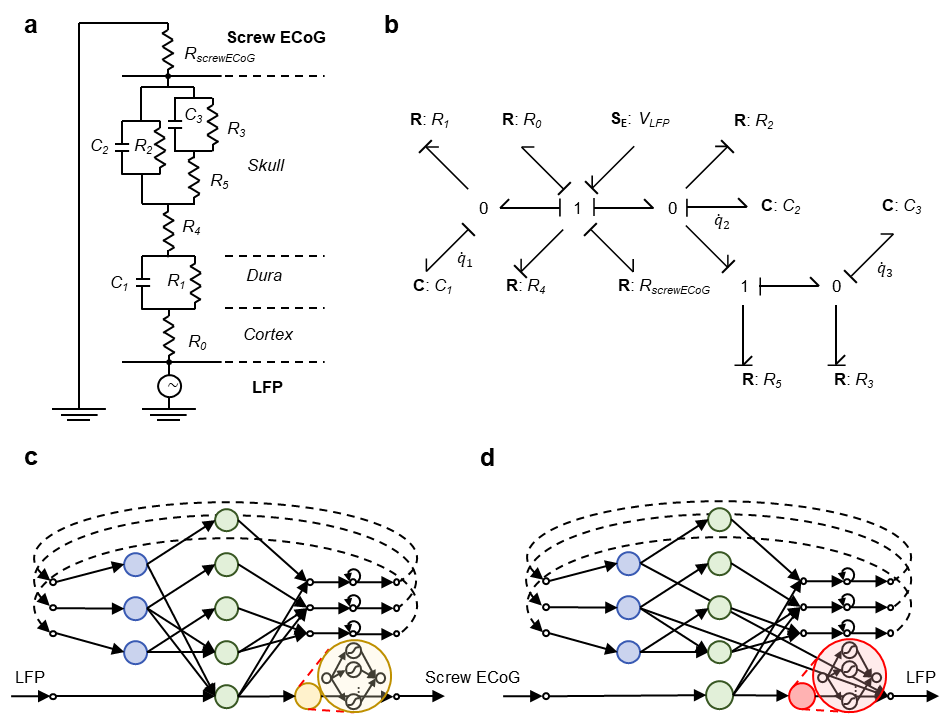
**

**Supplementary Figure 1: Development of the NBGNet.** (**a**) LFP-screw ECoG transmission electrical circuit was established based on the effective electrical signal pathway. (**b**) Bond Graph of the physical system illustrated in **a**. (**c-d**) Both forward- and inverse-NBGNet are derived from the system dynamics equations for the LFP-screw ECoG transmission model. (**c**) Schematic of forward-NBGNet architecture, where the colored circles represent a multi-layer perceptron unit. (**d**) Same as **c** for inverse-NBGNet architecture.


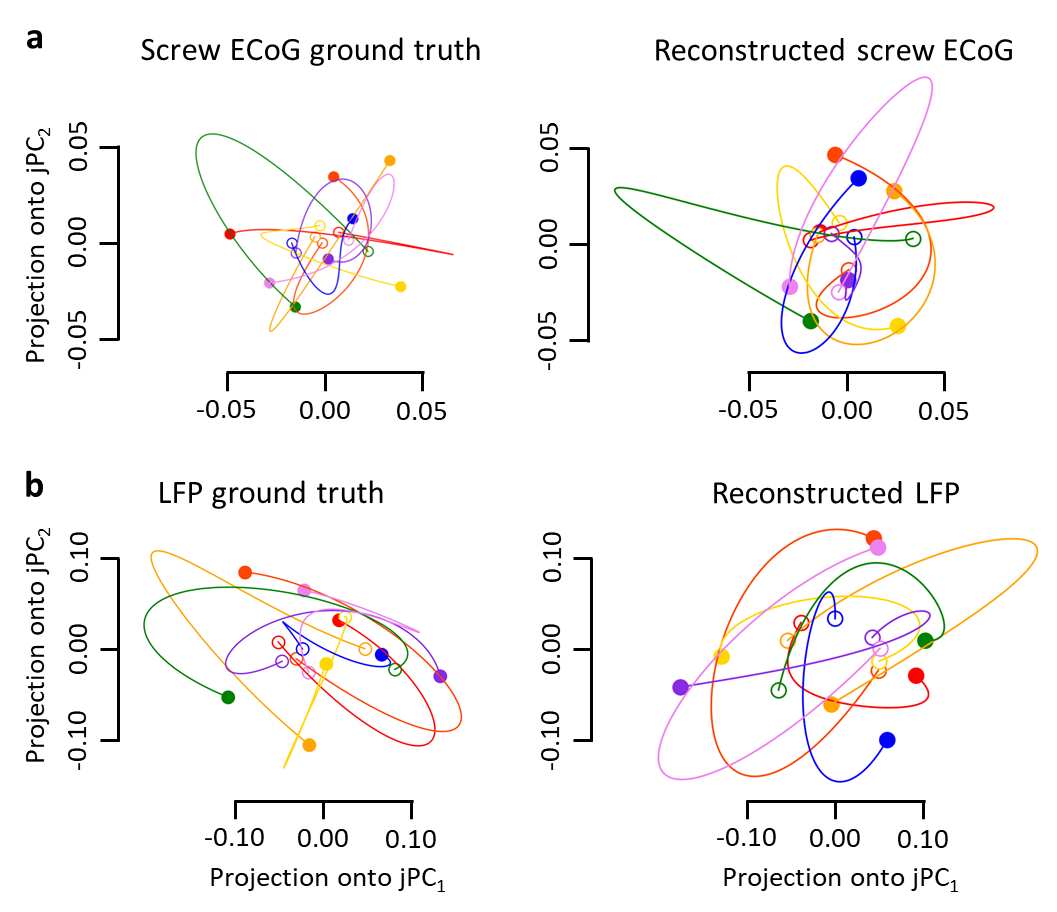


**Supplementary Figure 2: NBGNet captures and reconstructs rotational latent dynamics in the reaching-out task.** (**a**) Representative jPCA-derived latent trajectories from the ground-truth screw ECoG (left) and reconstructed screw ECoG (right). (**b**) Representative jPCA-derived latent trajectories from the ground-truth LFP (left) and reconstructed LFP (right). Each color represents each target direction in **Figure 5a**. Unfilled dots represent the onset of the latent trajectories, and the filled dots represent the endpoint of the latent trajectories.


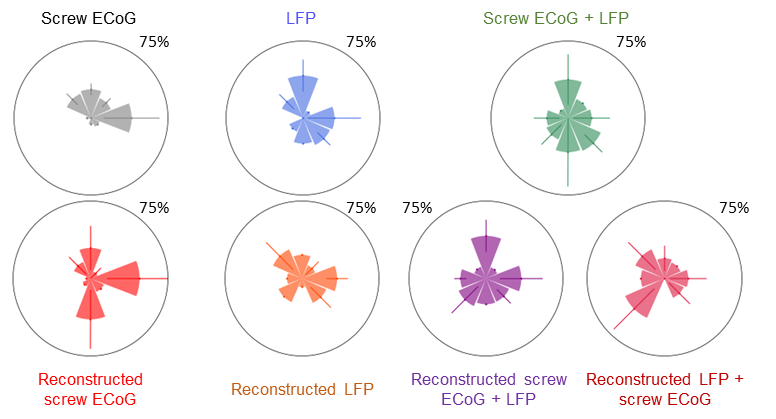


**Supplementary Figure 3: The classification accuracy with respect to each target direction.** Polar plots of the classification accuracy with respect to eight different target directions for LDA classifiers trained with seven different conditions (error bars, s.d.; n = 4). The color codes are the same as **Figure 6**.
